# Insights into AIM-InDel diversities in Yunnan Miao and Hani ethnic groups of China for forensic and population genetic purposes

**DOI:** 10.1186/s41065-022-00238-9

**Published:** 2022-05-20

**Authors:** Wei Cui, Shengjie Nie, Yating Fang, Man Chen, Ming Zhao, Qiong Lan, Chunmei Shen, Bofeng Zhu

**Affiliations:** 1 Guangzhou Key Laboratory of Forensic Multi-Omics for Precision Identification, School of Forensic Medicine, Southern Medical University, Guangzhou, 510515 China; 2 Microbiome Medicine Center, Department of Laboratory Medicine, Zhujiang Hospital, Southern Medical University, Guangzhou, 510282 China; 3 School of Forensic Medicine, Kunming Medical University, Kunming, 650500 China; 4 Department of Laboratory Medicine, Nanfang Hospital, Southern Medical University, Guangzhou, 510515 China; 5 Key Laboratory of Shaanxi Province for Craniofacial Precision Medicine Research, College of Stomatology, Xi’an Jiaotong University, Xi’an, 710004 China

**Keywords:** Ancestry informative marker, InDel, Population genetics, Yunnan Miao group, Yunnan Hani group

## Abstract

**Background:**

Ancestry informative markers are regarded as useful tools for inferring the ancestral information of an individual, which have been widely used in the criminal investigations and population genetic studies. Previously, a multiplex amplification panel containing 39 AIM-InDel loci was constructed. This study aims to investigate the genetic polymorphisms of these 39 AIM-InDel loci in Yunnan Hani and Miao ethnic groups, and to uncover their genetic affinities with reference populations based on the AIM-InDel markers.

**Materials and methods:**

In this research, 39 AIM-InDel profiles of 203 unrelated Miao individuals and 203 unrelated Hani individuals in Yunnan province of China were acquired. Additionally, we evaluated the genetic polymorphisms of 39 InDel loci in Yunnan Miao and Hani groups. Moreover, the genetic relationships among Yunnan Miao, Hani and reference populations were also clarified based on *Nei*’s genetic distances, pairwise fixation indexes, principal component analyses, phylogenetic analyses, and STRUCTURE analyses.

**Results:**

Genetic diversity analyses demonstrated that these InDel loci showed varying degrees of genetic polymorphisms, and could be utilized in forensic identifications in Yunnan Miao and Hani groups. The results of principal component analyses, phylogenetic analyses and Structure analyses revealed that Yunnan Miao and Hani groups had closer genetic relationships with East Asian populations, especially with the populations from Southern China. This research enriched the genetic data of Chinese ethnic minority, and provided ancestral information of Yunnan Miao and Hani groups from the perspective of population genetics.

**Supplementary Information:**

The online version contains supplementary material available at 10.1186/s41065-022-00238-9.

## Introduction

Ancestry inference of unknown DNA donors acquired from crime scene is important to narrow the scope of criminal investigation and uncover the genetic affinities among populations. Single nucleotide polymorphisms (SNPs), with the merits of low mutation rates, widespread distributions in human genome, small amplified fragments, various detection methods as well as relevance to human phenotypes and so on, have been the most common ancestry informative markers (AIMs) in the forensic genetics [1–3]. Insertion/deletion (InDel) polymorphisms, as a kind of length polymorphic marker, combine the superiorities of short tandem repeats (STRs) and SNPs such as short amplicon sizes, easy to detect on capillary electrophoresis (CE) platform and low mutation rates, which have been widely used in forensic human individual identifications (HID), ancestry inference and population genetics [4–6]. In recent decades, many sets of AIM-SNP panels and AIM-InDel panels have been constructed to analyze the ancestral origins or genetic patterns within or among continental populations using CE or massively parallel sequencing (MPS) technologies. Incipiently, intercontinental AIM panels mainly focused on specific intercontinental populations (such as populations from Africa, Europe and East Asia) [7–10]. In recent years, regional AIM panels which concentrated on the inference of ancestral origins in subpopulations or closely related populations have been widely constructed [11–14].

Yunnan province, bounded by Qinghai-Tibet Plateau in the northwest and the Yunnan-Guizhou Plateau in the east, is a mountainous province in the southwest part of China. The Hani nationality is one of the unique ethnic minorities in Yunnan province. According to the statistical data from the 6th census of China in 2010, there are more than 1.6 million Hani individuals living in Yunnan province [15]. Besides China, the Hani people also distribute in Vietnam, Burma, Thailand, Laos and other countries in Southeast Asia [16]. The language of Hani belongs to Yi language of Tibeto-Burman branch, Sino-Tibetan language family. Hani language has no characters previously, and now use Latin-based spelling characters [17].

With a population of greater than 9 million in China, Miao ethnic minority (or Hmong) is an ethnic group with long history residing in southwest part of China like Guizhou, Guangxi and Yunnan provinces. There are more than 1.2 million Miao individuals in Yunnan province, accounting for 12.76% of the total Miao individuals [15]. Besides China, the Miao people also live in Southeast Asian countries like Vietnam, Burma, and Laos. Linguists believe that Miao language belongs to Hmong-Mien family of languages [18]. The script of the Miao nationality has been lost, and Miao people in China use Latin-based spelling characters or Chinese.

Most of the population genetic studies on the Chinese Miao and Hani ethnic groups were limited to individual identification panels based on autosomal STRs, InDel markers, or Y-chromosomal and X-chromosomal STRs [16–20]. However, rare population genetic studies and ancestry analyses focusing on Chinese Miao and Hani groups have been conducted using ancestry informative markers, which make them insufficient for the genetic background researches of Chinese Miao and Hani groups. Previously, an AIM-InDel panel containing 39 InDel loci based on CE platform was constructed [21]. The results of developmental and efficacy validations of ancestry inference revealed that this panel was an efficient tool to predict the biogeographical origins for African, European, East Asian and Eurasian populations [22–24]. This study aims to investigate the genetic polymorphisms of these 39 AIM-InDel loci in Yunnan Hani and Miao ethnic groups, and to uncover their genetic affinities with reference populations based on these AIM-InDel markers.

## Methods and materials

### Ethical approval and sample collections

The study protocol has been reviewed, permitted, and supervised by the ethics committee of Xi’an Jiaotong University Health Science Center (Ethical Approval Number: 2019–1039). This research was conducted in accordance with the ethical principles for medical research involving human subjects recommended by the World Medical Association Declaration of Helsinki. A total of 406 bloodstain samples (Hani = 203, Miao = 203) were collected from Yunnan province, China. All volunteers were unrelated healthy Hani or Miao individuals and declared that their families lived in Yunnan for at least three generations. Every volunteer finished a questionnaire about personal information and health status, and then signed a written informed consent before sample collection. Peripheral venous blood was dried on FTA card and then stored at room temperature.

### Multiplex PCR amplification and allele genotyping

The 1.2 mm^2^ bloodstain was directly amplified by the multiplex PCR system of 39 AIM-InDel panel on GeneAmp PCR system 9700 (Thermo Fisher Scientific, Foster City, CA, USA). The components of PCR reagents and thermal cycle parameters were depicted in previous articles [22, 23]. Capillary electrophoresis of PCR products was conducted on the ABI 3500xL Genetic Analyzer (Thermo Fisher Scientific, Foster City, CA, USA). After capillary electrophoresis, GeneMapper ID-X (Thermo Fisher Scientific, Foster City, CA, USA) was used to determine the allelic genotypes of 39 AIM-InDel loci. During the multiple amplification and capillary electrophoresis, DNA 9948 and deionized water were used as positive and negative controls, respectively.

### Quality control

The whole experimentation was implemented in an accredited laboratory (Multi-Omics Innovative Research Center of Forensic Identification) by China National Accreditation Service for Conformity Assessment (CNAS). This AIM-InDel panel has already passed the developmental and internal validations [22].

### Reference populations

In the evaluations of genetic affinities between the studied groups and reference populations, the 38 out of 39 AIM-InDel loci were included because population data of rs3034941 were not available in 1000 Genome Project. Population data of the same 38 AIM-InDel loci in 30 reference populations selected from public database and published researches were included in the present study as reference database. Among these reference populations, 26 of which were acquired from 1000 Genome Project while population data of Chinese Qinghai Tibetan (CTQ), Chinese Tibet Tibetan (CTT), Chinese Kirgiz (CKX) and Chinese Uyghur (CUX) were acquired from previously published articles [21, 23, 25]. The detail information of the reference database was shown in Supplementary Table 1.

### Statistical analyses

The Fisher’s exact tests of Hardy-Weinberg equilibrium (HWE) for all loci were performed by the Arlequin software [26]. Forensic statistical parameters of 39 AIM-InDel loci in Yunnan Miao and Yunnan Hani groups including allelic frequencies, expected heterozygosity (He), polymorphism information content (PIC), matching probability (MP), observed heterozygosity (Ho), power of discrimination (PD), probability of exclusion (PE) and typical paternity index (TPI) were calculated using an online tool-STRAF, and then were visualized in the form of violin plots [27]. Linkage disequilibrium (LD) tests were calculated for all pairwise loci using SNPAnalyzer 2.0 software [28]. Genetic distances (*D*_*A*_) between Yunnan Hani (or Yunnan Miao) and other reference populations were calculated by DISPAN program [29]. Pairwise fixation index (*F*_*ST*_) and corresponding *p*-values between Yunnan Hani (or Yunnan Miao) and other reference populations were calculated by Arlequin v3.5 software [26], and then were visualized in the form of Nightingale rose diagram by *R* software. Phylogenetic analyses were conducted by different algorithms to evaluate the phylogenetic relationships among Hani, Miao and reference populations. A neighbor-joining (NJ) tree was conducted based on insertion allelic frequencies of AIM-InDel loci by Phylip 3.69 package [30], and then visualized with Mega 7 software [31]. We performed a series of distance-based TreeMix analyses between Yunnan Hani (or Yunnan Miao) and other reference populations. We constructed the maximum likelihood (ML) tree with ESN as the root population, and 0–10 mixture events were simulated to reconstruct the gene flow events [32]. Principal component analyses (PCA) at population and individual levels were performed by *R* software. Ancestry component analyses were conducted by STRUCTURE v2.3.4 software with predefined *K* values from 2 to 6 [33]. Admixture software [34] and CLUMPAK (http://clumpak.tau.ac.il/) online tools were used to further analyze and visualize the results of STRUCTURE.

## Results

### Genetic polymorphisms and forensic parameters of 39 AIM-InDel loci

#### Results of Hardy-Weinberg equilibrium and linkage disequilibrium tests

In the present study, rs5896844 locus was excluded in the analyses of HWE and LD since only deletion allele was found at this locus in Yunnan Hani and Miao groups. *P*-values of the Fisher’s exact tests of HWE for all loci in Yunnan Hani and Miao groups were listed in Supplementary Tables 4 and 5. And no significant deviations from HWE were observed in these two groups. The *r*^*2*^ values for pairwise loci in the LD analyses were shown in Supplementary Tables 2 and 3. All pairwise loci were confirmed to linkage equilibrium both in Yunnan Hani and Miao groups except for rs3033760 and rs36038238 loci (Yunnan Miao: *r*^*2*^ = 0.5312; Yunnan Hani: *r*^*2*^ = 0.4948).

#### Allelic frequencies and forensic statistical parameters of 39 AIM-InDel loci

**Fig. 1:**
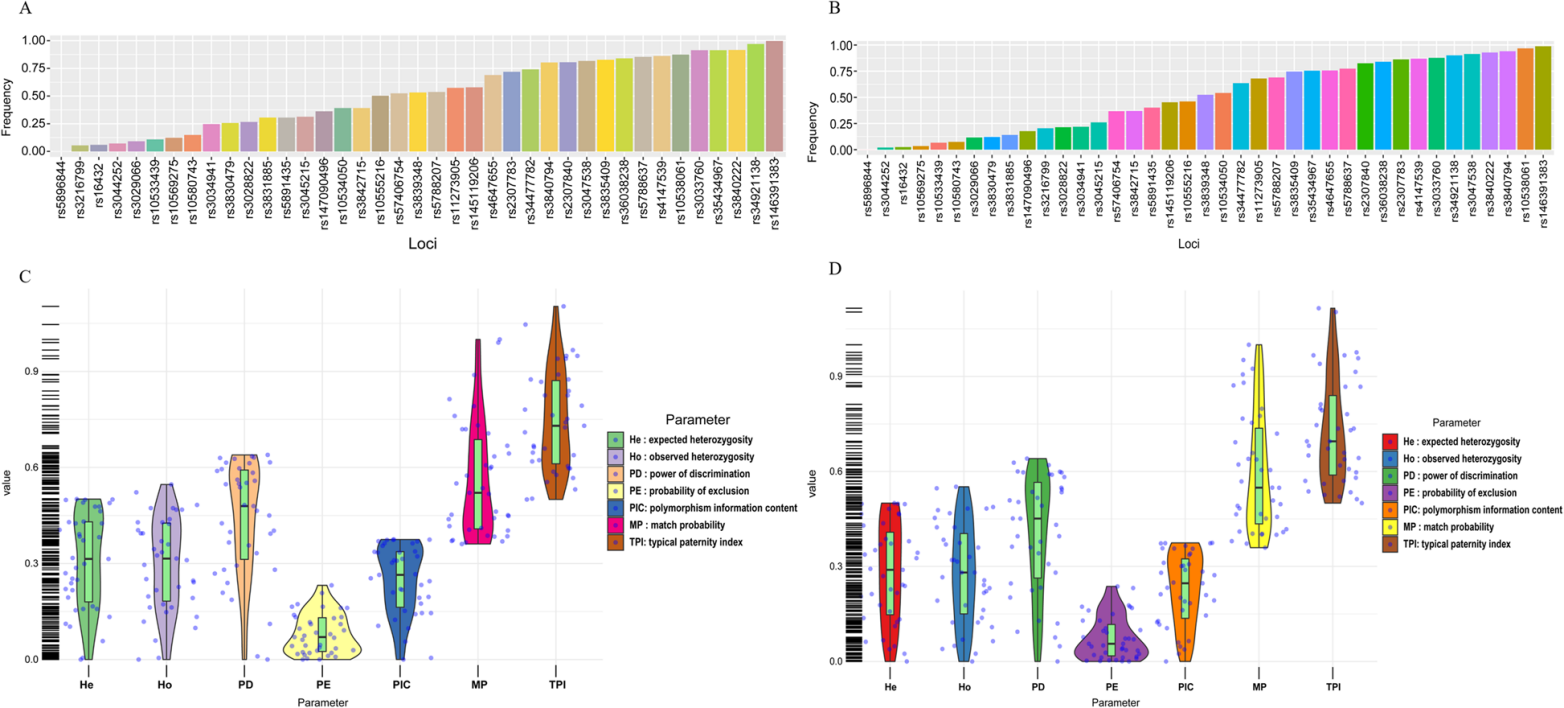
Results of allelic frequencies and forensic parameters of 39 AIM-InDel loci in Yunnan Miao and Hani groups. **A** and **B** displayed the bar plots of insertion allelic frequencies of 39 InDel loci in Yunnan Hani and Miao groups, respectively. **C** and **D** showed the violin plots of forensic parameters of 39 AIM-InDel loci in Yunnan Hani and Miao groups, respectively

Allelic frequencies of 39 AIM-InDel loci as well as their forensic statistical parameters were calculated in Yunnan Hani and Miao groups, respectively, and the results were shown in Fig. 1, Supplementary Tables 4 and 5. In this section, forensic parameters of rs5896844 locus were not evaluated because only deletion allele was observed in the two studied groups. When we calculated the CPD and CPE of this panel in Yunnan Hani and Miao groups, rs3033760 was eliminated due to the relevance between rs3033760 and rs36038238. Figure 1A and C displayed the bar plots of insertion allelic frequencies and violin plots of forensic statistical parameters of 39 AIM-InDel loci in Yunnan Hani group, respectively. The insertion allelic frequencies of 39 AIM-InDel loci ranged from 0 (rs5896844) to 0.9975 (rs146391383). For 39 AIM-InDel loci, the rs10555216 displayed the largest values of expected heterozygosity and polymorphism information content, while rs57406754 showed the largest values of observed heterozygosity and expected heterozygosity. Besides, the maximum value of power of discrimination was observed at rs11273905 in the studied Yunnan Hani group. The combined power of discrimination (CPD) and probability of exclusion (CPE) of 37 AIM-InDel loci (rs3033760 and rs5896844 were excluded) were 0.9999999999617927 and 0.96457903 in Yunnan Hani group, respectively.

For Yunnan Miao group, bar plots of insertion allelic frequencies and violin plots of forensic statistical parameters of 39 AIM-InDel loci were shown in Fig. 1B and D. The insertion allelic frequencies of 39 AIM-InDel loci in Yunnan Miao group ranged from 0 (rs5896844) to 0.9877 (rs146391383). For the forensic parameters of 39 AIM-InDel, the maximum values of expected heterozygosity and polymorphism information content were found at rs3839348. The combined power of discrimination and probability of exclusion of 37 InDel loci were 0.999999999557958 and 0.954507431 in Yunnan Miao group, respectively.

### Population genetic analyses among Yunnan Miao, Hani and reference populations

#### Population differentiation analyses between the studied groups and reference populations

**Fig. 2:**
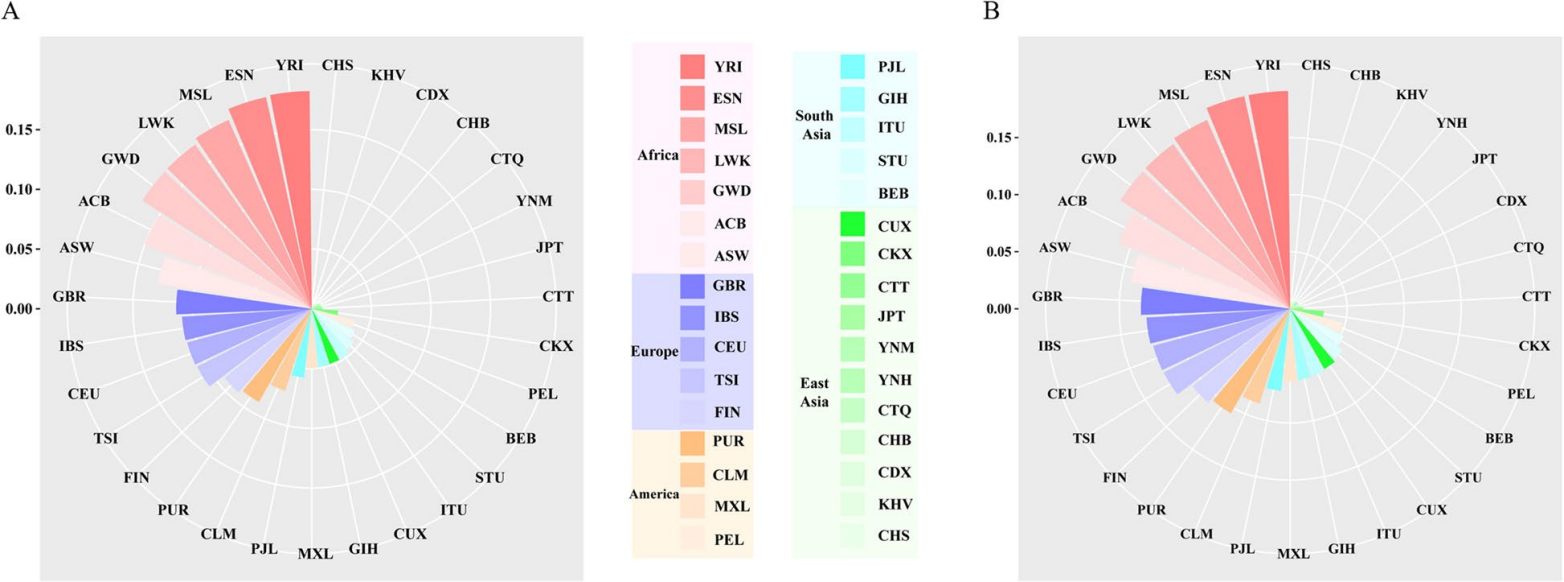
The *D*_*A*_ values were calculated to measure the genetic differentiations between the studied groups and reference populations. **A** showed the *D*_*A*_ values between Yunnan Hani group and reference populations. **B** showed the *D*_*A*_ values between Yunnan Miao group and reference populations. Reference populations: seven populations from Africa including African Caribbean in Barbados (ACB), African Ancestry in Southwest US (ASW), Esan in Nigeria (ESN), Gambian in Western Division (GWD), Luhya in Webuye, Kenya (LWK), Mende in Sierra Leone (MSL) and Yoruba in Ibadan (YRI); five populations from South Asian including Gujarati Indian in Houston (GIH), Indian Telugu in the UK (ITU), Sri Lankan Tamil in the UK (STU), Punjabi in Lahore (PJL) and Bengali in Bangladesh (BEB); nine populations from East Asia including Chinese Dai in Xishuangbanna (CDX), Han Chinese in Beijing (CHB), Southern Han Chinese (CHS), Chinese Qinghai Tibetan (CTQ), Chinese Tibet Tibetan (CTT), Chinese Kirgiz (CKX), Chinese Uyghur (CUX), Kinh in Ho Chi Minh City, Vietnam (KHV) and Japanese in Tokyo (JPT); five populations from Europe including Utah residents with Northern and Western European ancestry (CEU), Finnish in Finland (FIN), British in England and Scotland (GBR), Iberian populations in Spain (IBS) and Toscani in Italy (TSI); four populations from America groups including Colombian in Medellin (CLM), Colombia, Mexican Ancestry in Los Angeles (MXL), Peruvian in Lima (PEL) and Puerto Rican in Puerto Rico (PUR)

The *D*_*A*_ values and pairwise *F*_*ST*_ values were calculated to measure the genetic differentiations between the studied groups and 30 reference populations. The *D*_*A*_ distances between the Hani, Miao and reference populations were shown in Fig. 2A and B, respectively. For Hani group, the smallest *D*_*A*_ value was found between Hani and Southern Han Chinese (CHS, *D*_*A*_ = 0.0037), followed by Kinh in Ho Chi Minh City Vietnam (KHV, *D*_*A*_ = 0.0040) and Chinese Dai in Xishuangbanna (CDX, *D*_*A*_ = 0.0041), as shown in Fig. 2A. For Miao group, the nearest genetic distance was observed between Miao and CHS (0.0056), followed by Han Chinese in Beijing (CHB, *D*_*A*_ = 0.0068) and KHV (0.0076), as shown in Fig. 2B. Pairwise *F*_*ST*_ values between the studied groups and reference populations were displayed in Supplementary Table 6. For Hani group, the smallest *F*_*ST*_ value was found between Hani and CHS (*F*_*ST*_ = 0.0094, *p* < 0.00001), followed by CDX (*F*_*ST*_ = 0.0101, *p* < 0.00001) and KHV (*F*_*ST*_ = 0.0104, *p* < 0.00001), whereas the largest *F*_*ST*_ values were found between Hani and African populations. For Miao group, the smallest *F*_*ST*_ value was observed between Miao and CHS (*F*_*ST*_ = 0.0212, *p* < 0.00001), followed by CHB (*F*_*ST*_ = 0.0253, *p* < 0.00001) and KHV (*F*_*ST*_ = 0.0277, *p* < 0.00001) groups.

#### Allelic frequency distributions of 38 InDel loci among the studied groups and 30 reference groups

**Fig. 3:**
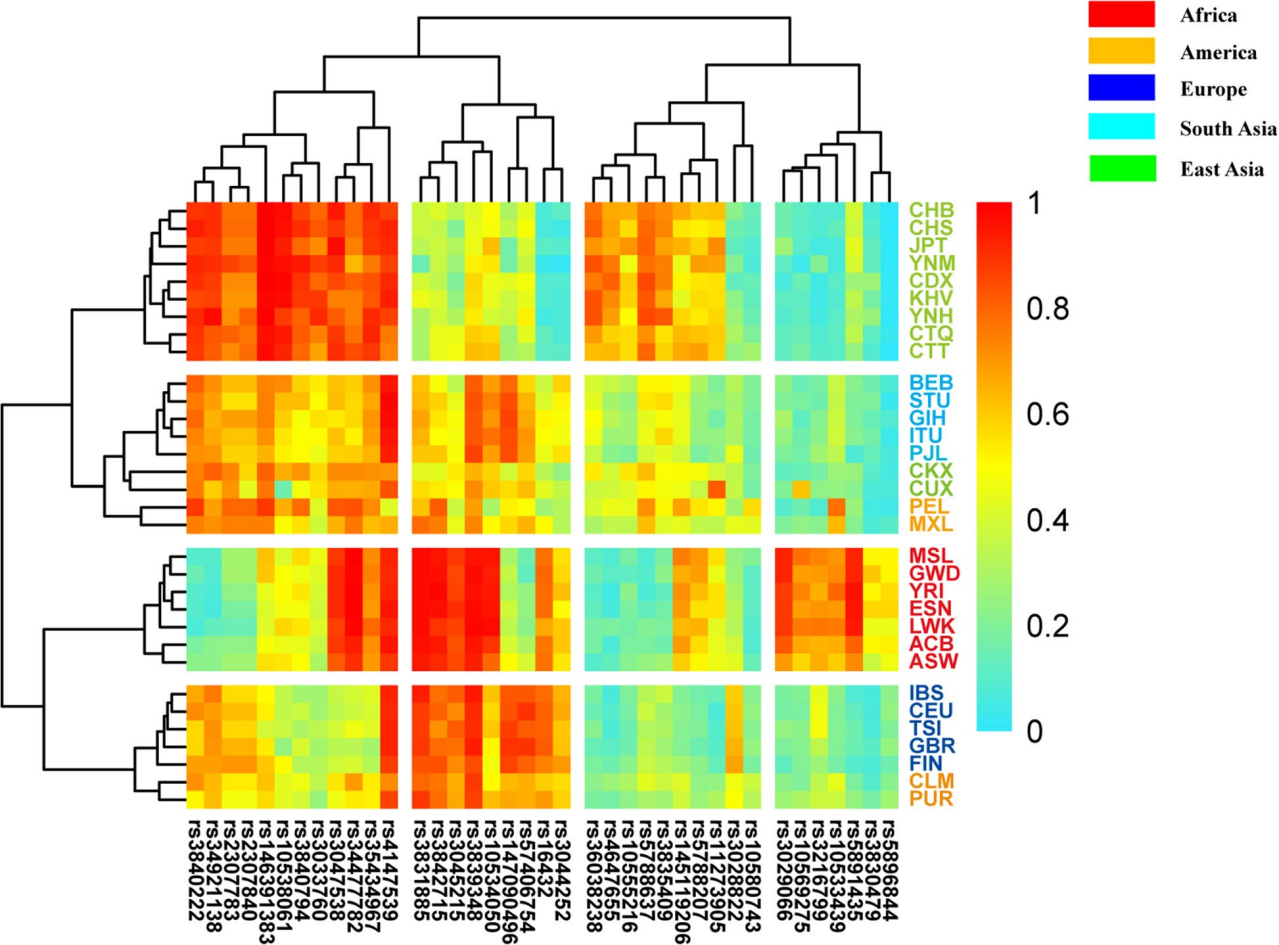
A heatmap showed the allelic frequency distributions of 38 AIM-InDel loci in the studied groups and reference populations

A heatmap was conducted to evaluate the insertion allelic frequency distributions of 38 AIM-InDel loci in Yunnan Miao, Hani and reference populations, and the plot was shown in Fig. 3. Allele frequency differential (δ) values of AIM-InDel loci in pairwise intercontinental populations were also calculated, and the results were shown in Supplementary Table 7. For δ values, there were 25 AIM-InDel loci with the δ values greater than 0.4 between African and East Asian populations. Thirteen AIM-InDel loci showed the δ values greater than 0.4 between African and European populations. Nineteen AIM-InDel loci showed the δ values greater than 0.4 between East Asian and European populations.

For the cluster analyses of InDel loci, four main clusters could be identified: (1) loci rs3033760, rs10538061, rs146391383, rs2307840, rs2307783, and rs3840222 displayed large insertion allele frequencies in East Asian populations and large δ values between African and East Asian populations; (2) rs3831885, rs3842715, and rs3045215 displayed high insertion allelic frequencies in African and European populations. The δ values of these three loci were greater than 0.5 between African and East Asian populations, and greater than 0.4 between European and East Asian populations; (3) loci rs36038238, rs4647655, rs5788637 and rs3835409 displayed relative high frequencies in East Asian populations but low frequencies in African and European populations. The δ values of these four loci were greater than 0.5 between East Asian and African populations, and were greater than 0.39 between East Asian and European populations; (4) loci rs3029066, rs10569275, rs3216799, rs10533439 and rs5891435 displayed relative high insertion allelic frequencies in African populations but relative low frequencies in non-African populations. Large δ values of most InDel loci were observed among African and non-African populations. The largest δ value of rs5891435 locus was found between African and European populations.

Cluster analyses for both InDel loci and populations were conducted, and the results were shown in Fig. 2. Thirty-two populations clustered into four different clusters based on the allelic frequency distributions of 38 InDel loci: (1) the studied Yunnan Miao and Hani groups clustered together with the populations from East Asia. The Yunnan Miao, CHB, Japanese in Tokyo Japan (JPT) and CHS groups clustered in the same subclade while Hani group clustered closely with CDX and KHV groups; (2) seven populations from Africa clustered together in the same branch; (3) five populations from Europe displayed the similar frequency distributions of 38 AIM-InDel and they clustered in the same subclade; (4) five populations from South Asia clustered together.

#### Phylogenetic analyses of reference populations and the two studied groups

**Fig. 4:**
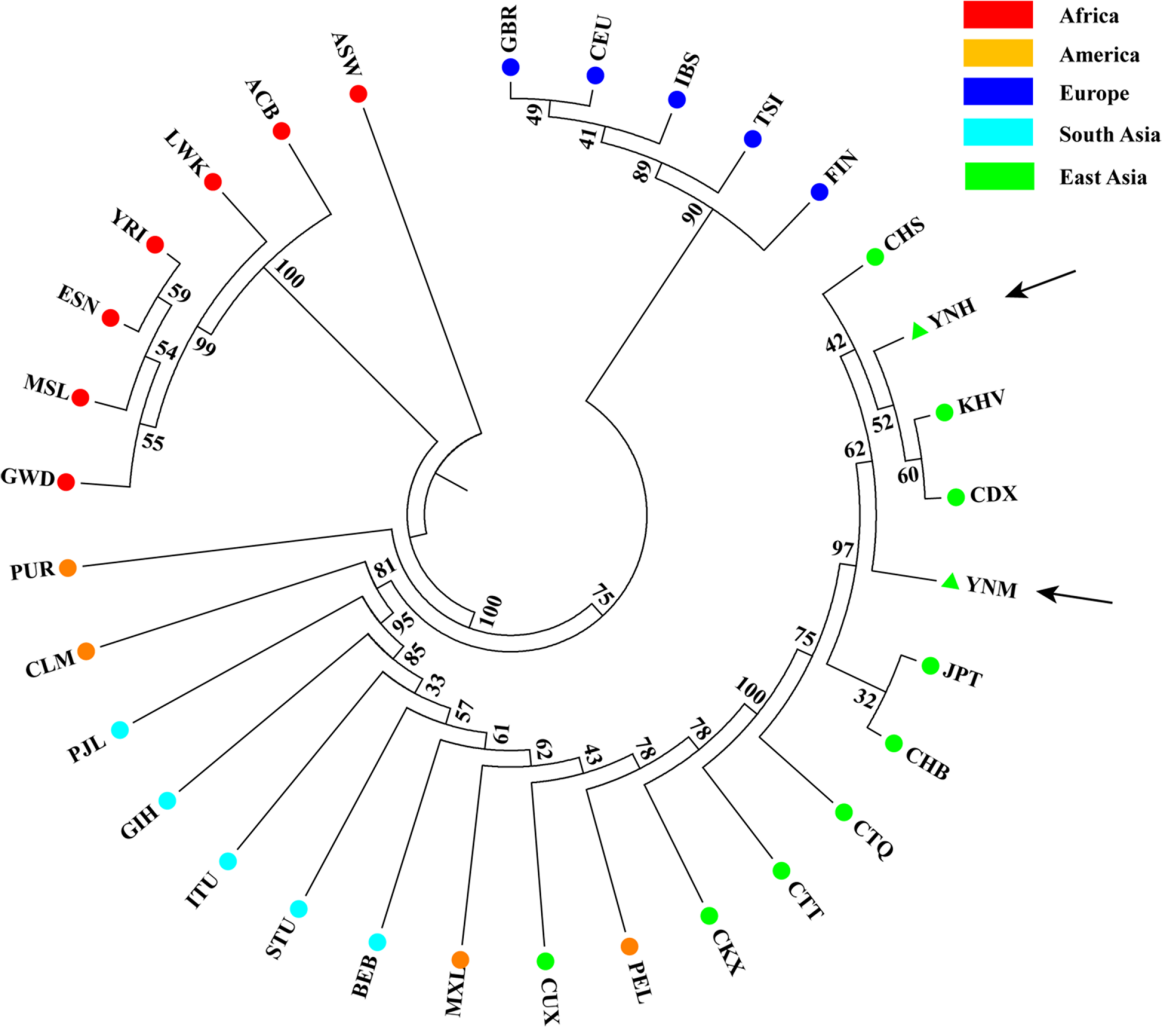
A neighbor-joining tree was conducted based on insertion allelic frequencies of 38 AIM-InDel loci by Phylip package, and then visualized with Mega 7 software. (The arrows point to the two studied groups)

Phylogenetic analyses of 32 groups based on the same AIM-InDel loci were conducted using NJ and ML methods. Overall speaking, all 32 groups could be divided into four main clusters according to their intercontinental locations (African, European, East Asian and South Asian populations) in NJ tree (Fig. 4). The Yunnan Hani and Miao groups clustered together with the populations from East Asia. The Yunnan Hani clustered closely with CDX and KHV groups while Miao group clustered closely with CHS group.

**Fig. 5:**
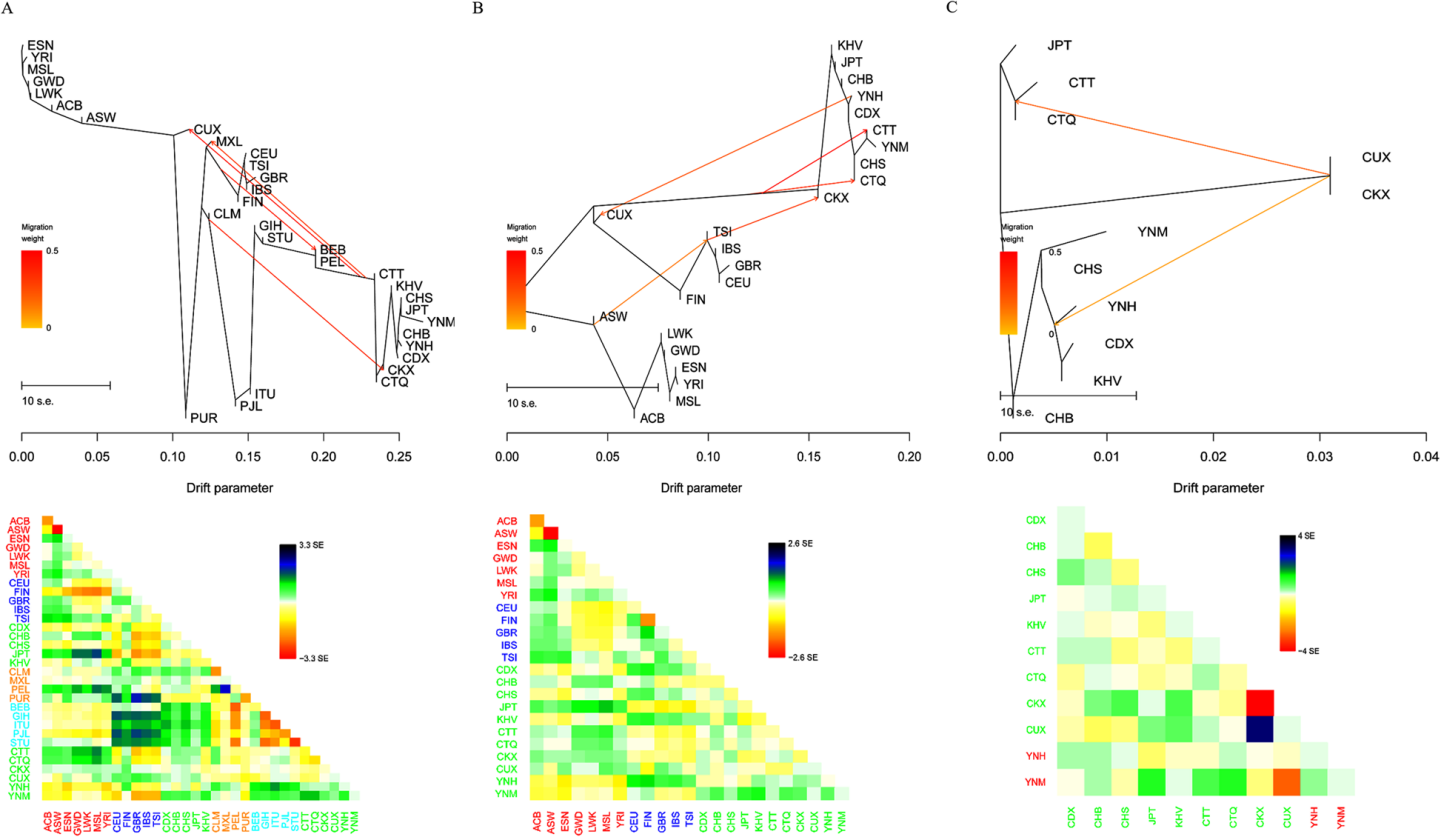
Phylogenetic trees showed the phylogenetic relationships among Yunnan Miao, Hani and reference populations by maximum likelihood (ML) method. **A** ML tree for 32 populations assumed that four migration events happened. **B** ML tree for populations from Africa, Europe and East Asia assumed that four migration events happened. **C** ML trees for populations from East Asia assumed that two migration events happened. Reference populations: seven populations from Africa including African Caribbean in Barbados (ACB), African Ancestry in Southwest US (ASW), Esan in Nigeria (ESN), Gambian in Western Division (GWD), Luhya in Webuye, Kenya (LWK), Mende in Sierra Leone (MSL) and Yoruba in Ibadan (YRI); five populations from South Asian including Gujarati Indian in Houston (GIH), Indian Telugu in the UK (ITU), Sri Lankan Tamil in the UK (STU), Punjabi in Lahore (PJL) and Bengali in Bangladesh (BEB); nine populations from East Asia including Chinese Dai in Xishuangbanna (CDX), Han Chinese in Beijing (CHB), Southern Han Chinese (CHS), Chinese Qinghai Tibetan (CTQ), Chinese Tibet Tibetan (CTT), Chinese Kirgiz (CKX), Chinese Uyghur (CUX), Kinh in Ho Chi Minh City, Vietnam (KHV) and Japanese in Tokyo (JPT); five populations from Europe including Utah residents with Northern and Western European ancestry (CEU), Finnish in Finland (FIN), British in England and Scotland (GBR), Iberian populations in Spain (IBS) and Toscani in Italy (TSI); four populations from America groups including Colombian in Medellin (CLM), Colombia, Mexican Ancestry in Los Angeles (MXL), Peruvian in Lima (PEL) and Puerto Rican in Puerto Rico (PUR)

To further evaluate the phylogenetic relationships from different population scales, we performed genetic distance-based ML trees among Yunnan Hani, Miao and reference populations. We constructed the ML trees with ESN as the root population firstly, and 0–10 mixture events were gradually added to construct the migration events. As shown in Fig. 5A, ML tree for 32 populations with assuming four migration events happened was constructed. Seven populations from Africa clustered together as root populations. Five populations living in Europe clustered together and located in the middle of the ML tree. Eight populations from China, JPT and KHV clustered together as the East Asian cluster. The studied Yunnan Hani and Miao groups clustered closely with East Asian populations. ML trees for populations from Africa, Europe and East Asia with assuming four migration events happened were constructed, and the phylogenetic tree was shown in Fig. 5B: seven populations from Africa clustered together as root populations, which located in the bottom part of the ML tree; five populations from Europe clustered closely, and located in the middle of the tree; eight populations from China, JPT and KHV groups clustered together as the East Asian cluster; Utah residents with Northern and Western European Ancestry (CEU), a Eurasian population located between East Asian and European populations; the studied Yunnan Hani and Miao groups clustered closely with East Asian populations. ML trees for East Asian populations were also conducted (Fig. 5C), and we found that the studied Yunnan Hani and Miao groups clustered closely with CHS, CDX and KHV groups.

#### Principal component analyses at different population scales

**Fig. 6:**
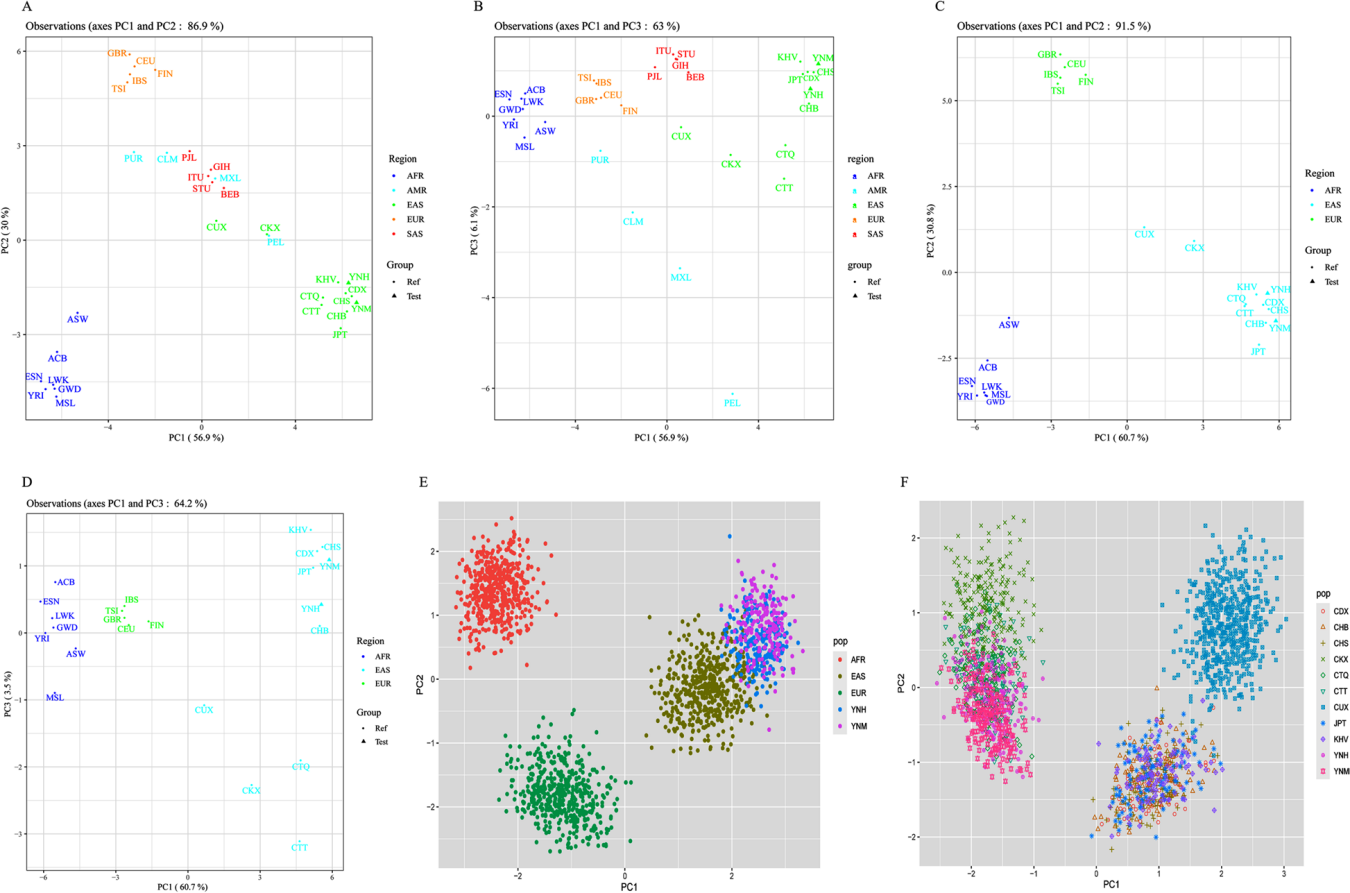
Principal component analyses (PCA) at different population scales. **A** and **B** PCA plots among 32 reference populations based on PC1 and PC2, PC1 and PC3, respectively. **C** and **D** PCA among 23 populations from Africa, Europe and East Asia based on PC1 and PC2, PC1 and PC3, respectively. **E** Population data of African, European and East Asian groups acquired from 1000 Genome Project were used as reference data to construct a PCA plot in individual level. **F** Population data of East Asian groups (acquired from 1000 Genome Project and published articles) were used as reference data to construct a PCA plot at individual level. (Abbreviation: AFR, Africa; EAS, East Asia; EUR, Europe)

Genetic affinities of Yunnan Hani, Miao and reference populations based on the allelic frequencies of 38 AIM-InDel loci were also conducted by performing PCA at different population scales. PCA plots among 32 worldwide populations based on PC1 and PC2, PC1 and PC3 were conducted, and the plots were shown in Fig. 6A and B, respectively. The top three components could explain a total of 93% variation. PC1 could successfully distinguish the African, European and East Asian populations from the rest populations. The studied Yunnan Miao and Hani placed adjacent to East Asian populations in the PC1 and PC2.

To further analyses the genetic relationships on a fine scale among Yunnan Hani and Miao groups, the PCA plots among 23 population from African, European and East Asian populations were conducted, and the plots were shown in Fig. 6C and D, respectively. The first three components could explain a total of 95% variation. PCA plots showed that seven populations from Africa clustered together and located on the lower left part of the plot; five populations from Europe located on the top of the plot; nine populations from East Asia clustered together in the lower right part of the plot while two groups from Eurasia (CUX and CKX groups) located between East Asian and European populations; and the studied Yunnan Hani and Miao groups placed adjacent to East Asian populations, especially groups located in southwest part of China.

In Fig. 6E, population data of African, European and East Asian populations acquired from 1000 Genome Project were used as reference populations to construct a PCA plot in individual scale. Individuals from Africa, Europe and East Asia clustered into three clusters, respectively. Individuals from Yunnan Miao (purple dots) and Hani (blue dots) placed more adjacent to East Asian populations. In Fig. 6F, population data of East Asian populations (acquired from 1000 Genome Project and previously published articles) were used as reference populations to construct a PCA plot at individual level. The results showed that individuals from Yunnan Miao and Hani groups overlapped with those from Chinese Tibetan.

#### Structure analyses of the studied Yunnan Hani and Miao groups

**Fig. 7:**
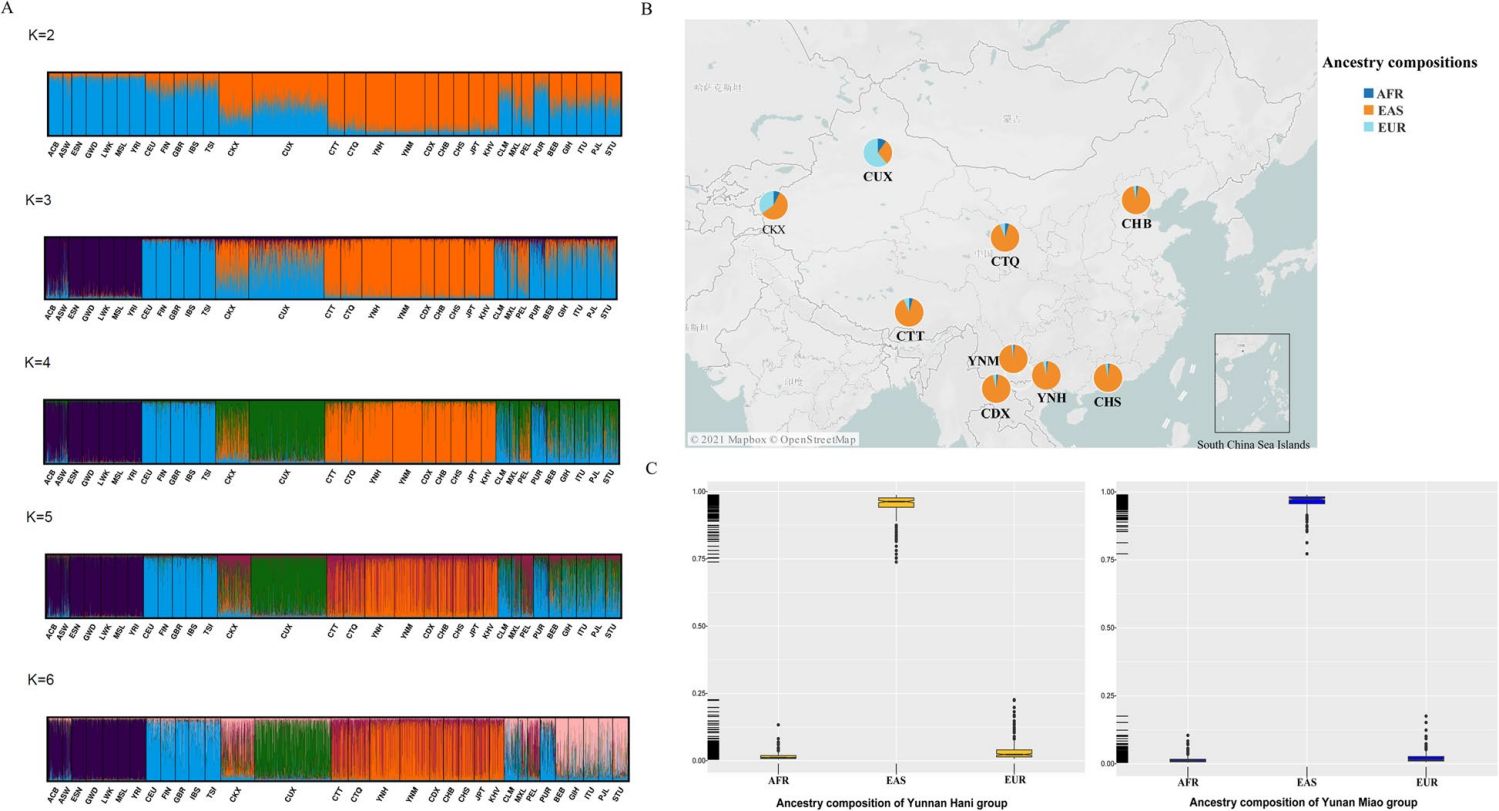
**A** Structure analyses among 3917 individuals using the Admixture model based on the raw genotypes under *K* values from 2 to 6. **B** Geographical locations of Yunnan Miao, Hani groups as well as reference groups in China. Pie charts represented the ancestral components of Yunnan Hani, Miao and other Chinese groups at *K* = 3. Blue, orange and cyan represented the African, East Asian and European ancestral components, respectively. **C** Boxplots showing the ancestral components of Yunnan Hani and Miao groups. (AFR: Africa; EAS: East Asia; EUR: Europe)

To further explore the genetic background of the studied Yunnan Hani and Miao groups, ancestral component estimations for Yunnan Hani and Miao groups were conducted firstly by STRUCTURE software via admixture model. Cross-validation (CV) error results calculated by ADMIXTURE suggested that the three-source model with the smallest CV value (0.64215) could be used to explain the genetic variations of continental populations. At *K* = 3, populations from Africa, Europe, East Asia and Eurasia were apparently distinguished from each other, and identified by purple, cyan, orange and a combination color of cyan and orange, respectively. East Asian-specific ancestral component (orange) was maximized in the Yunnan Hani and Miao groups (as shown in Fig. 7A). When four ancestral components were assumed, Eurasian-specific ancestral component (green) was separated from populations in Africa, East Asia and Eurasia. With the increase of *K* values, no obvious substructures within intercontinental populations were observed. Ancestral components of Yunnan Hani, Miao and other groups in China were calculated (as shown in Fig. 7B and C). The studied Yunnan Hani and Miao groups mainly composed of the East Asian-based ancestral compositions (with the averages of 94.6 and 96.3%, respectively), and shared similar ancestry compositions with groups in Southern China.

## Discussion

The development of economy and society promotes the continuous communications among people from different regions. Frequent gene exchanges among Yunnan Miao, Hani and neighboring populations make their genetic relationships become closer, and also make their gene pools carry the genetic traits of other ethnic groups to some extent. Exploring the genetic feature and genetic structure of Yunnan Miao and Hani groups are of great significance for understanding the population genetic background of the Chinese nation. In the present study, we evaluated the genetic polymorphisms of 39 InDel loci in Yunnan Miao and Hani groups. Moreover, this research aimed to clarify the genetic relationships among Yunnan Miao, Hani and reference populations based on *Nei’s* genetic distances, pairwise fixation indexes, principal component analyses, phylogenetic analyses, and STRUCTURE analyses.

Before we calculated the forensic statistical parameters of 39 AIM-InDel loci in Yunnan Hani and Miao groups, Fisher’s exact tests of HWE for all the loci in Yunnan Hani and Miao groups were performed, and the results demonstrated that all loci (except for rs5896844) reached Hardy-Weinberg equilibrium both in Yunnan Hani and Miao groups. According to the genotype data of rs5896844 in 1000 Genome Project Phase III (GRCh38.p13) and dbSNP (build 155), the locus rs5896844 was a diallelic InDel marker. The deletion allele frequencies of rs5896844 in African, European and East Asian populations were 0.4800, 0.7890 and 0.9960, respectively. The genetic polymorphisms were decreasing from Africa to East Asia accordingly. This might be due to bottlenecks in the history of the non-African populations [35]. In the current study, the rs5896844 was excluded to conduct the test of HWE because only deletion allele was observed in the Yunnan Miao and Hani groups.

LD analyses were used to evaluate the relevance between pairwise loci. In this study, rs5896844 locus was excluded to conduct pairwise LD analyses because only deletion allele was observed in the Yunnan Miao and Hani groups. The results of LD analyses showed that the rs3033760 and rs36038238 were observed the strong LD, which might result from the close position between rs3033760 (Chr 3:173839871–173,839,881) and rs36038238 (Chr 3:173820325–173,820,328) (about 0.019546 cM).

The CPD and CPE values of 37 AIM-InDel loci demonstrated that these InDel loci showed high discriminability for individual identifications in Yunnan Hani and Miao groups, though linkage disequilibrium between rs3033760 and rs36038238 was observed. Population genetic researches for Chinese Miao or Hani groups were also evaluated based on different InDel panels. Jiang et.al found that the CPD and CPE values of 21 InDel loci in Miao group were 0.999998008541708 and 0.85884504, respectively [36]. Chen et.al calculated the CPD and CPE values in Guizhou Miao group (CPD = 0.99999999998, CPE = 0.9884) based on 30 InDel loci of Investigator DIPplex kit [37]. Huang et.al calculated the CPD and CPE values of 17 STR loci in Yunnan Hani group. They found the CPD value of 17 STR loci in Yunnan Hani group was higher than 0.999999999 and the CPE value was 0.999999792 [38]. In comparisons with these panels, the individual identification efficiency of 37 InDel loci was comparable to that of 17 STR loci [38], which indicated that this panel could be used in individual identifications in Yunnan Miao and Hani groups. However, there was limit efficiency in the parentage testing due to relative low CPE values in two studied groups.

The ancestry components of Yunnan Hani and Miao groups, as well as the genetic relationships among Yunnan Hani, Miao and reference populations were also investigated. Phylogenetic analyses showed that Yunnan Miao and Hani groups had close relationships with East Asian populations, especially with groups from Southern China (CHS and CDX). A set of PCA plots at different population scales were conducted to analyze the genetic relationships between the studied groups and reference populations, confirming that this AIM-InDel panel could be an effective tool for distinguishing East Asian, European and African populations. At individual level, it was demonstrated that Yunnan Miao and Hani individuals had closer ties with East Asian populations, especially with the groups living in Southern China, compared with other intercontinental populations. These PCA results were also in accordance with the results of pairwise *D*_*A*_, *F*_*ST*_ values and phylogenetic analyses. Estimations for ancestral components of Yunnan Hani and Miao groups using admixture model demonstrated that the ancestry components of Yunnan Miao and Hani groups were dominated by East Asian ancestry components.

According to the Miao’s myths and legends handing down from generation to generation, the ancestor of Miao group is Chi You. Around 5000 years before present, the ancestors of the Miao people migrated to the middle reach of the Yangtze River because of the wars and conflicts (http://www.chinadaily.com.cn/culture/art/2014-06/17/content_17591898_6.htm). During the long history, part of the Miao ancestors gradually migrated to the Southern and Western regions of China, and entered the mountainous areas of Southwest China and Yunnan-Guizhou Plateau. During Qin and Han Dynasties (from about 221 B.C. to 220 A.D.), Miao people mainly settled in Guizhou, Hunan and Hubei provinces (Southern China). After that, there were close and frequent genetic and cultural exchanges among Miao, Yao, Han and other groups in Southwest regions of China [39, 40], which resulted in closer genetic distances and similar ancestry components between Miao and Southern Chinese groups. Population genetic analyses based on autosomal InDel loci and X-STR loci also found that Chinese Miao groups residing in different regions are genetically closer related to the adjacent populations, which supported our results [18, 20].

Many scholars believed that the ancestors of Hani group were the ancient Qiang nationality who lived in Qinghai-Tibet Plateau [41]. During Qin Dynasty (around 200 B.C.), ancient Qiang nationality left Qinghai-Tibet Plateau and migrated to Southwest region of China. In the Tang Dynasty, a group of the ancient Qiang people migrated west to the basin between the Yuanjiang River and the Lancang River, and settled there, becoming the ancestors of the modern Hani people [41]. The prolonged settlement with Yunnan Yi, Southern Han, Dai and other groups together made closer genetic distances among Yunnan Hani and neighboring groups. Population genetic researches based on different genetic markers revealed that the Yunnan Hani group had close genetic relationships with groups living in the Southern China [16, 17, 38]. The present results were in accordance with the published researches.

## Conclusions

In summary, genetic polymorphisms of the 39 InDel loci in Yunnan Miao and Hani groups were assessed. Most of InDel loci were high polymorphisms, and could be utilized in forensic individual identifications in Yunnan Miao and Hani groups. Moreover, population genetic analyses revealed that Yunnan Miao and Hani groups had closer genetic relationships with East Asian populations, especially with the groups from Southern China.

## Supplementary Information

**Additional file 1: Supplementary Table 1.** List of reference populations and their abbreviations, sample sizes and locations. **Supplementary Table 2. ***r*^2^ values, *x*^2^ and their *p*-values for pairwise loci in the linkage disequilibrium analyses (Yunnan Hani group). **Supplementary Table 3. ***r*^*2*^ values, *x*^2^ and their *p-*values for pairwise loci in the linkage disequilibrium analyses (Yunnan Miao group). **Supplementary Table 4.** Forensic parameters of 39 AIM-InDel loci as well as their *p* values for Hardy-Weinberg equilibrium tests in Yunnan Miao ethnic group of China (*n* = 203). **Supplementary Table 5.** Forensic parameters of 39 AIM-InDel loci as well as their *p* values for Hardy-Weinberg equilibrium tests in Yunnan Hani ethnic group of China (*n* = 203). **Supplementary Table 6.** The *F*_*ST*_ values between the studied groups and reference populations. **Supplementary Table 7.** Allele frequency differential (δ) of 38 AIM-InDel loci in pairwise intercontinental populations.

## Data Availability

The raw genotype data used and analyzed during the current study are available from the corresponding author on reasonable request.
